# Author Correction: Non-consecutive enzyme interactions within TCA cycle supramolecular assembly regulate carbon-nitrogen metabolism

**DOI:** 10.1038/s41467-024-50599-0

**Published:** 2024-07-23

**Authors:** Weronika Jasinska, Mirco Dindo, Sandra M. C. Cordoba, Adrian W. R. Serohijos, Paola Laurino, Yariv Brotman, Shimon Bershtein

**Affiliations:** 1 Department of Life Sciences, Ben-Gurion University of the Negev, Beer-Sheva, Israel; 2 Protein Engineering and Evolution Unit, Okinawa Institute of Science and Technology Graduate University, Okinawa, Japan; 3 Max-Planck-Institut fur Molekulare Pflanzenphysiologie, Potsdam-Golm, Germany; 4 Departement de Biochimie, Universite de Montreal, Quebec, Canada; 5 Centre Robert-Cedergren en Bio-informatique et Genomique, Universite de Montreal, Quebec, Canada; 6 Institute for Protein Research, Osaka University, Suita, Japan; 7 Present Address: Department of Medicine and Surgery, Section of Physiology and Biochemistry, University of Perugia, Perugia, Italy

**Keywords:** Metabolomics, Multienzyme complexes, Molecular biophysics

Correction to: *Nature Communications* 10.1038/s41467-024-49646-7, published online 20 June 2024

The original version of this Article contained an error in the Acknowledgements section. The correct version of the Acknowledgements is:

We are grateful to Alisdair Fernie, Eugene Shakhnovich, and Barak Rotblat for the useful discussions and comments on the manuscript. We thank Gudrun Walter for their help in acquisition of GC-MS data. S.B. was supported by the Israel Science Foundation personal research grant 593/21. P.L. was supported by the Okinawa Institute of Science and Technology Graduate University (OIST) with subsidy funding from the Cabinet Office, Government of Japan. M.D. thanks the financial support from Japan Society for the Promotion of Science (JSPS) for the Kakenhi Early Career Scientist N.22K15065.

This has been corrected in the PDF and HTML versions of the Article.

