# Supplementary material for: Non-consecutive enzyme interactions within TCA cycle supramolecular assembly regulate carbon-nitrogen metabolism
Source: Nat Commun. 2024 Jun 20;15:5285. doi: 10.1038/s41467-024-49646-7 (PMC11189929; doi:10.1038/s41467-024-49646-7)
Supplement: Supplementary file 1 — Supplementary Information [file 41467_2024_49646_MOESM1_ESM.pdf]

Supplementary Information

Non-consecutive enzyme interactions within TCA cycle supramolecular assembly regulate carbon-nitrogen metabolism

Weronika Jasinska[1], Mirco Dindo[2,3], Sandra M.C. Cordoba[4], Adrian W.R. Serohijos[5,6], Paola Laurino[3*], Yariv Brotman[1*] and Shimon Bershtein[1*]

[1]Department of Life Sciences, Ben-Gurion University of the Negev, Beer-Sheva, Israel.
[2]current address: Department of Medicine and Surgery, Section of Physiology and Biochemistry, University of Perugia, Perugia, Italy
[3]Protein Engineering and Evolution Unit, Okinawa Institute of Science and Technology Graduate University, Okinawa, Japan
[4]Max-Planck-Institut fur Molekulare Pflanzenphysiologie, Potsdam-Golm, Germany
[5]Departement de Biochimie, Universite de Montreal, Quebec, Canada
[6]Centre Robert-Cedergren en Bio-informatique et Genomique, Universite de Montreal, Quebec, Canada
These authors contributed equally: Weronika Jasinska, Mirco Dindo
*Correspondence: Paola Laurino (paola.laurino@oist.jp), Yariv Brotman (brotmany@bgu.ac.il), Shimon Bershtein (shimonb@bgu.ac.il).

This PDF file includes:

Supplementary Fig. 1 to 20
Supplementary Tables 1 to 3
Supplementary Notes: Detailed Theory

Supplementary Figures

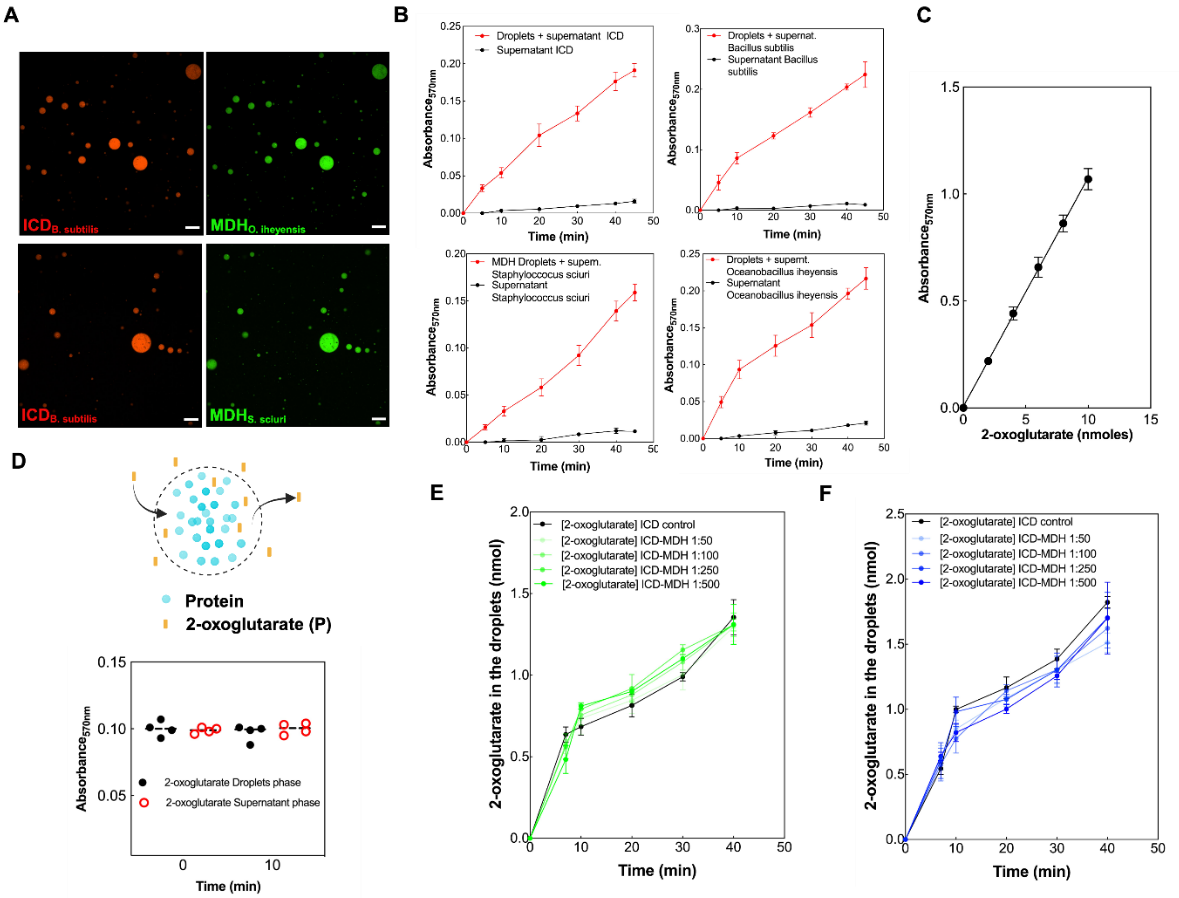

Supplementary Figure 1**. Partitioning and analysis of the interaction between Bacillus subtilis ICD and orthologues MDHs. (A)** Co-localization inside liquid-liquid phase separated protein droplets of ICD and orthologues MDHs from *S. sciuri* and *O. iheyensis*. ICD from *Bacillus subtilis* has been used 120 nM within the droplets and labeled with Alexa Fluor 594. MDHs from orthologues have been used 600 nM within the droplets and labeled with Alexa Fluor 488. Confocal images have been registered using a Nikon Spinning Disk. Scale bars are 20 µm. **(B)** Evaluation of enzyme partitioning within the droplet phase based on activity measurements. The products of the ICD and MDH reactions were measured using commercial kits as reported in the Material and Method section. **(C)** Calibration curve for the 2-oxoglutarate used for its quantification within the droplets and supernatant phases. **(D)** Evaluation of the partitioning of 2-oxoglutarate in the droplet and supernatant phases over time (10 minutes). Supplementary Figure 1D, *upper panel*, was created with BioRender.com, released under a Creative Commons Attribution-NonCommercial-NoDerivs 4.0 **(E)** An increase in ICD-MDH ratio within droplets does not cause an increase in delay of 2-oxoglutarate diffusion into the continuous phase. **(F)** An increase in ICD-MDH ratio within droplets does not cause an increase in delay of 2-oxoglutarate diffusion into the continuous phase. Data in all panels are represented as mean values ± SD from three independent experiments (n = 3). Source data to generate this figure are provided as a Source Data file.

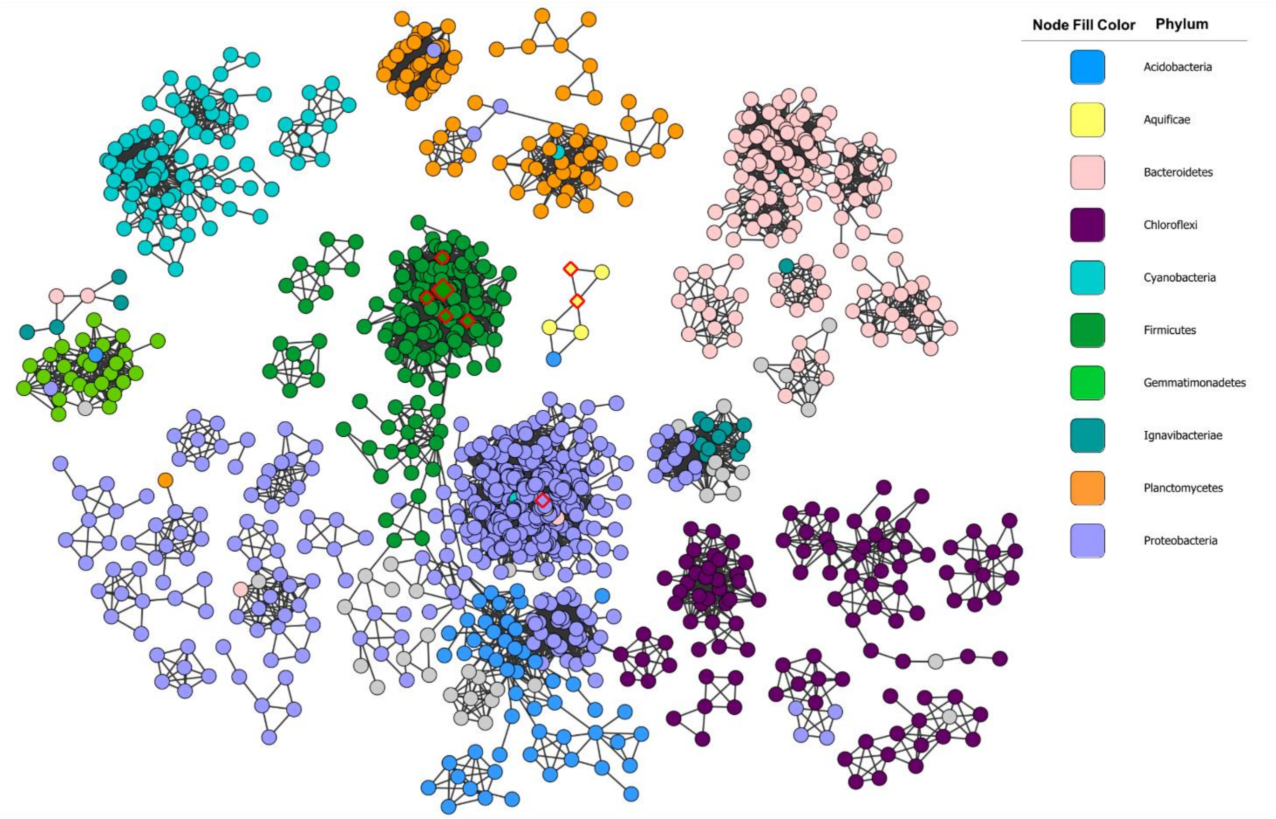

Supplementary Figure 2**. Amino-acid sequence similarity network of MDH from bacterial kingdom.** Nodes are colored according to a phylum. Nodes with diamonds contain the species MDH solubility and expression from which were experimentally tested. The biggest diamond node (dark green) is represented by *B. subtilis*, *Geobacillus kaustophilus*, *Oceanobacillus iheyensis*, and *Halobacillus halophilus*. Three other diamond nodes (dark green) from Firmicutes are: *Staphylococcus sciuri*, *Paenibacillus polymyxa*, and *Brevibacillus brevis*. One diamond node (light purple) from Proteobacteria represents *Brucella suis* and two diamond nodes (yellow) represent *Aquifex aeoliticus*.

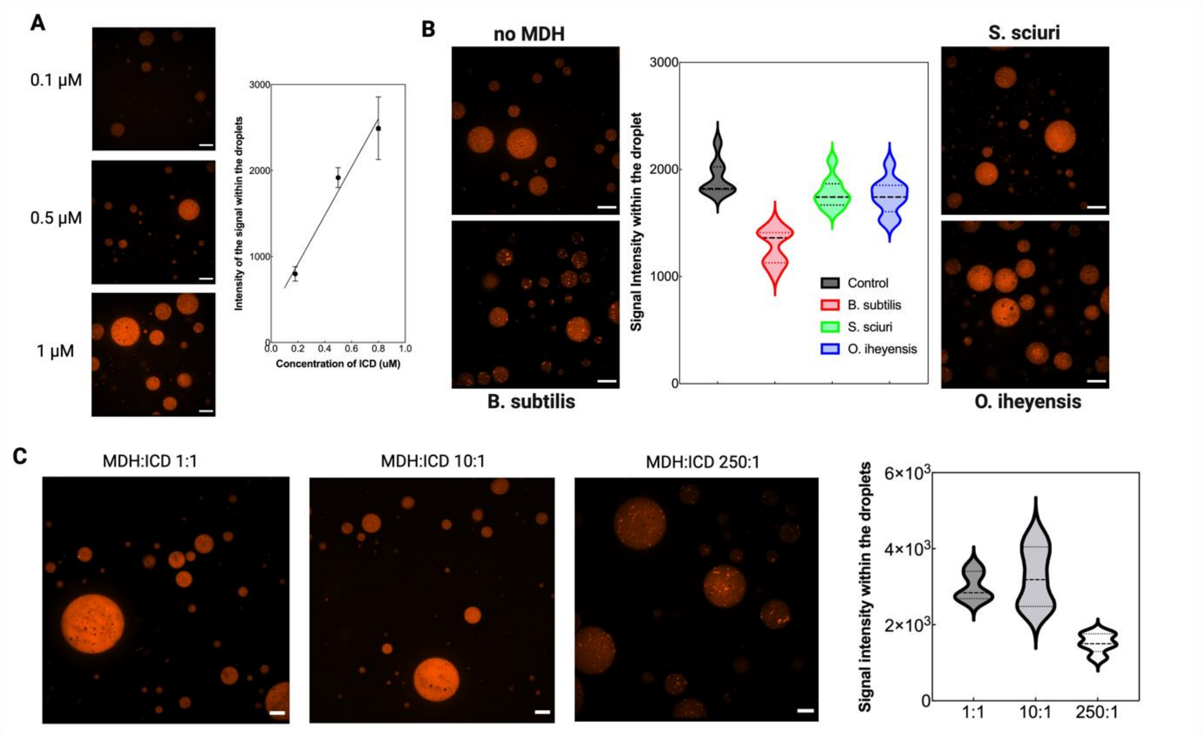

Supplementary Figure 3. **Qualitative analysis of ICD clustering mediated by MDH. (A)** Confocal images and calibration curve of the signal intensity mediated by Alexa Fluor 594 labeled ICD at three different concentrations (0.1 µM, 0.5 µM and 1µM). Data are represented as mean values ± SD from three independent experiments (*n* = 3). Scale bars are 20 µm. **(B)** Confocal images of ICD and analysis of the fluorescence intensity inside phase-separated droplets containing *B. subtilis* ICD only or coupled with a large excess of the three different MDHs employed in this study. ICD concentration within the droplets is around 2-2.5 µM while MDH concentration is around 400-500 µM. **(C)** Similar analysis at lower *B. subtilis* MDH:ICD ratios. 1:1 (2.5 µM ICD : 2.5 µM MDH), 10:1 (2.5 µM ICD : 25 µM MDH), 250:1 (2.5 µM ICD : 500 µM MDH) Scale bar is 20 µm. At least 5-10 droplets for each condition have been analyzed. Data are represented as mean values ± SD from three independent experiments (*n* = 3). Source data to generate this figure are provided as a Source Data file.

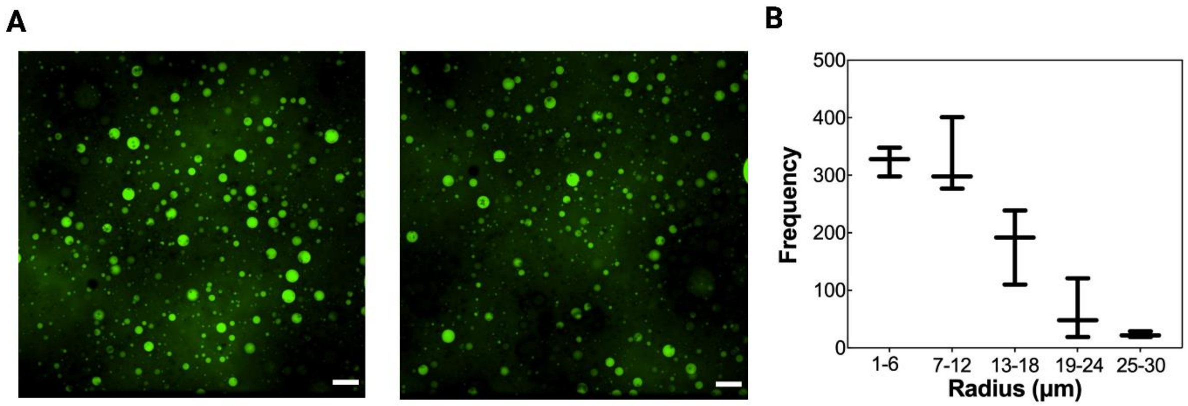

Supplementary Figure 4**. Size Distribution of BSA droplets. (A)** z-projections of single field of view of confocal z-stack for BSA droplets which have been diluted 1 time in supernatant. Confocal z-stacks were acquired using a Nikon Spinning Disk, using a 40X objective immediately after sample preparation, over a range of 100 μm from the bottom of the sample. **(B)** Radii distribution of BSA droplets diluted 1 time in supernatant. Horizontal bars represent mean values ± SD from for three independent experiments (*n* = 3). Scale bars are 25 μm. Source data to generate this figure are provided as a Source Data file.

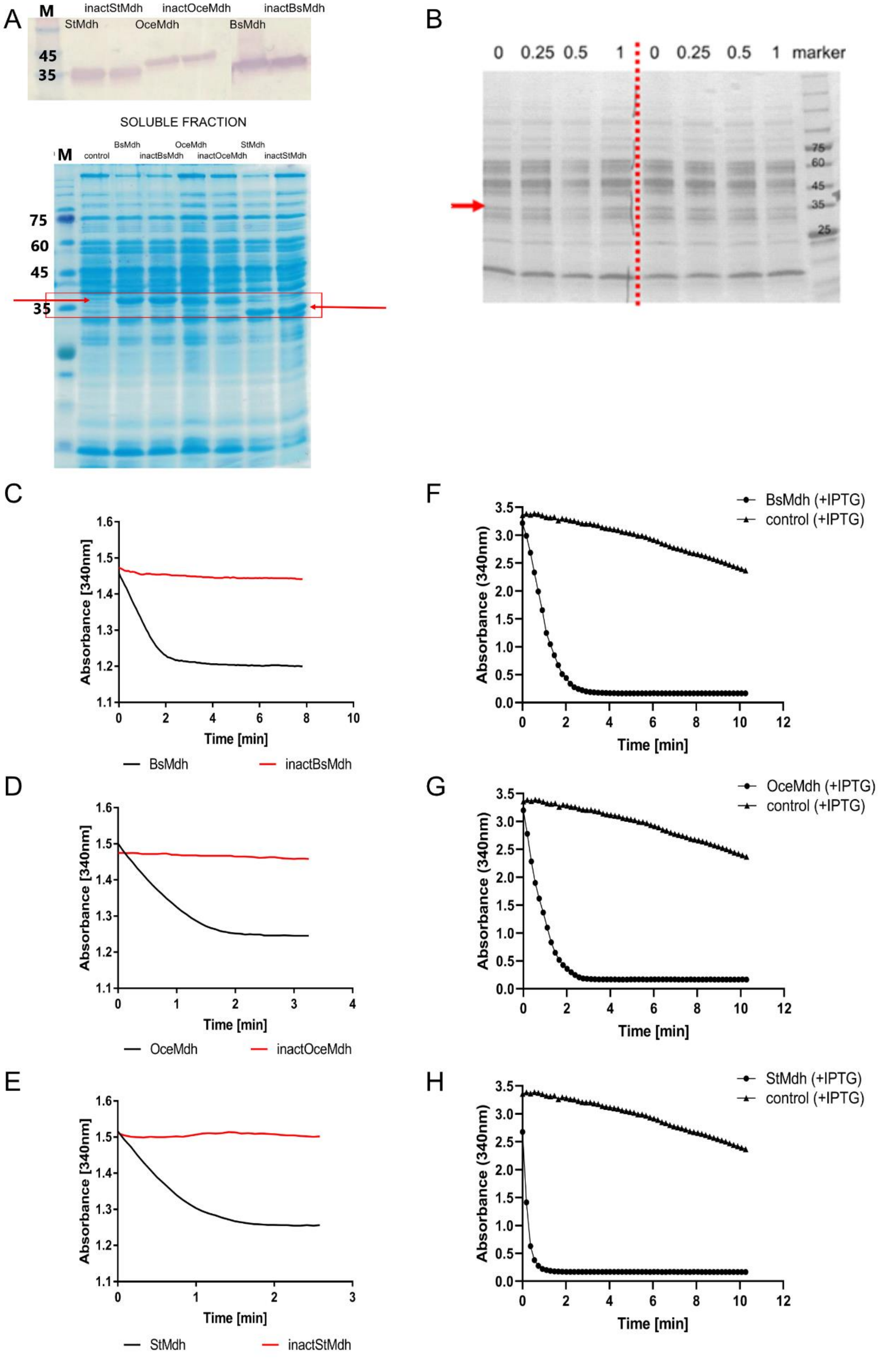
A
M
inactStMdh
inactOceMdh
inactBsMdh
StMdh
OceMdh
BsMdh
45
35
SOLUBLE FRACTION
M
control
BsMdh
inactBsMdh
OceMdh
inactOceMdh
StMdh
inactStMdh
75
60
45
35
B
0 0.25 0.5 1 0 0.25 0.5 1 marker
75
60
45
35
25
C
Absorbance [340nm]
Time [min]
BsMdh
inactBsMdh
D
Absorbance [340nm]
Time [min]
OceMdh
inactOceMdh
E
Absorbance [340nm]
Time [min]
StMdh
inactStMdh
F
BsMdh (+IPTG)
control (+IPTG)
Absorbance (340nm)
Time [min]
G
OceMdh (+IPTG)
control (+IPTG)
Absorbance (340nm)
Time [min]
H
StMdh (+IPTG)
control (+IPTG)
Absorbance (340nm)
Time [min]

Supplementary Figure 5**. Expression, solubility, and catalytic activity of MDH proteins. (A)** *Upper panel*. Western blot analysis of lysates shown in the *lower panel* with anti-His antibodies. *Lower panel*. SDS-PAGE Coomassie staining of the soluble fractions of *B. subtilis* cell lysates expressing MDH proteins from IPTG-induced pHT254 vector. Bands corresponding to MDH proteins are marked with a red rectangular frame and arrows. Note the approximately equal abundance of the soluble MDH proteins. M, protein marker, kDa. **(B)** Lack of visible MDH overexpression with IPTG-inducible $P_{hypersank}$ promoter placed upstream to *mdh* coding gene within *amyE* locus of the chromosome[1] is shown for comparison with (A). The first 4 lanes (up to the dashed red line) depict cell lysates extracted from WT *B. subtilis* strain. The lanes after the red dashed line show cell lysates extracted from *B. subtilis* cells with IPTG -induced expression of MDH from $P_{hypersank}$ promoter. The location of the anticipated MDH overexpression is marked with a red arrow. Numbers on top of the gel indicate IPTG levels in mM. **(C-E)** Comparison of the catalytic activities of purified active and inactive endogenous and orthologous MDH proteins (see Materials and Methods). Note the full inactivation of mutant MDHs. **(F-H)** Measurements of MDH activity in cell lysates expressing MDH proteins from IPTG-induced pHT254 vector and in cell lysates carrying empty pHT254 vector (in the latter case the MDH activity is originating solely from chromosomally encoded MDH). BsMDH/inactBsMDH, active/inactive *B. subtilis* MDH; StMDH/inactStMDH, active/inactive *S. sciuri* MDH*;* OceMDH/inactOceMDH, active/inactive *O. iheyensis* MDH; control, *B. subtilis* carrying empty pHT254 vector. Source data to generate this figure are provided as a Source Data file.

1. Guiziou, S. et al. A part toolbox to tune genetic expression in Bacillus subtilis. *Nucleic Acids Res* **44**, 7495-7508 (2016).

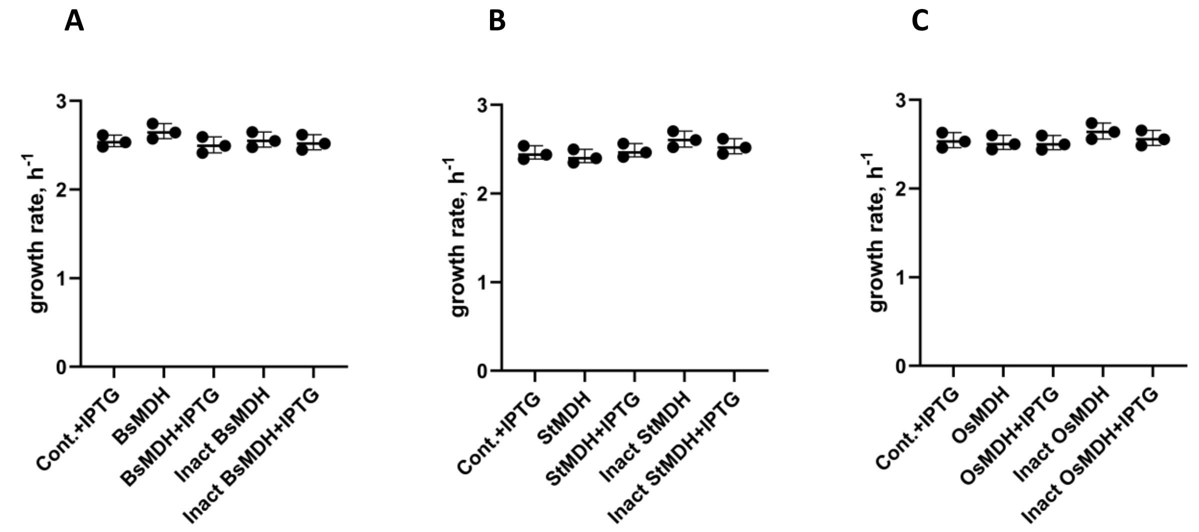

Supplementary Figure 6**. (A-C) Growth rates of *B. subtilis* strains expressing endogenous and orthologous MDH proteins from either induced (+IPTG) or uninduced pHT254 vector**. Horizontal bars represent mean values of three independent experiments (*n* = 3), whiskers indicate the spread of individual values from minimum to maximum. Individual values are shown as dots. BsMDH/inactBsMDH, active/inactive *B. subtilis* MDH; StMDH/inactStMDH, active/inactive *S. sciuri* MDH*;* OceMDH/inactOceMDH, active/inactive *O. iheyensis* MDH; control, *B. subtilis* carrying empty pHT254 vector. Source data to generate this figure are provided as a Source Data file.

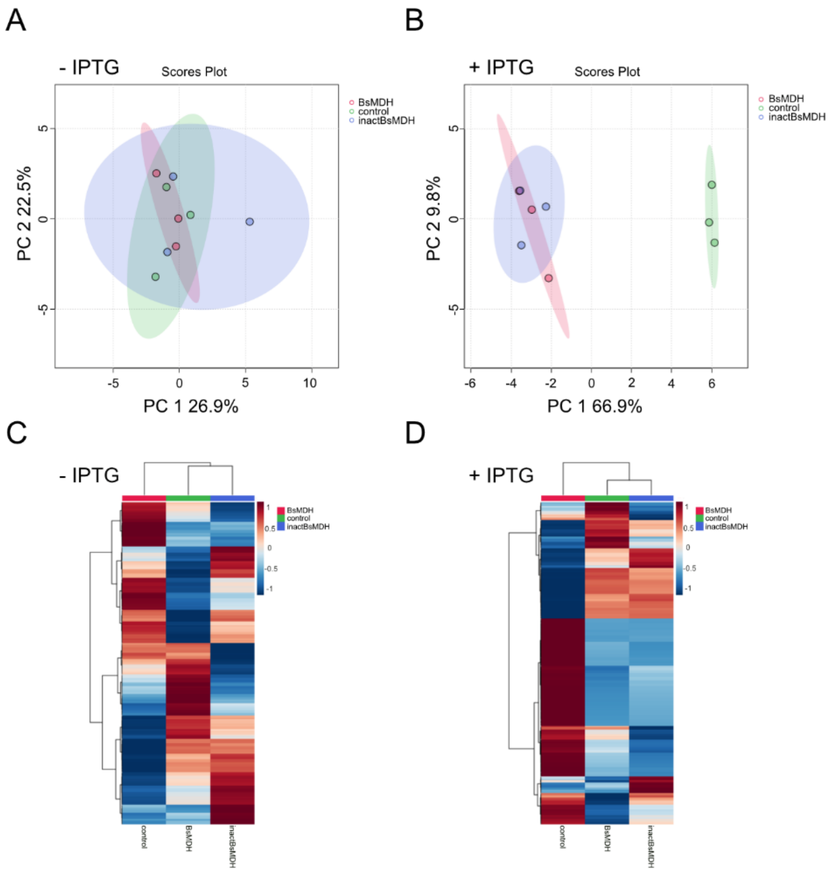

Supplementary Figure 7**. (A,B)** PCA of untargeted metabolite intensities identified in three *B. subtilis* replicate populations expressing active or inactive endogenous MDH proteins from either uninduced (A) or IPTG-induced (B) pHT254 vector. Note the separation on the PC1 axis between *B. subtilis* replicate populations expressing active/inactive endogenous MDH proteins and control populations (carrying empty pHT254 vector) upon IPTG induction. **(C,D)** Heat map of $-\log_2$ of relative intensities of untargeted metabolites in uninduced (C) and IPTG-induced *B. subtilis* populations. BsMDH/inactBsMDH, B. subtilis cells expressing active/inactive *B. subtilis* MDH; control, *B. subtilis* carrying empty pHT254 vector. Source data to generate this figure are provided as a Supplementary Data file 2.

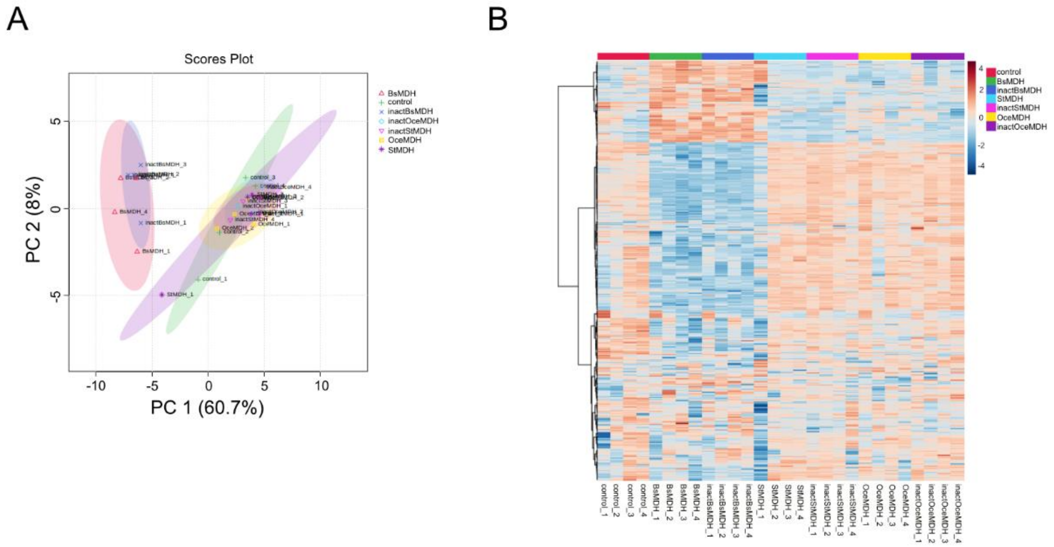

Supplementary Figure 8**. (A)** PCA of untargeted metabolite intensities identified in four *B. subtilis* replicate populations expressing either active or inactive endogenous and orthologous MDH proteins and control populations from IPTG-induced pHT254 vector. Note the separation on the PC1 axis between *B. subtilis* replicate populations expressing active/inactive endogenous MDH proteins and the rest of the populations **(B)** Heat map of $-\log_2$ of relative intensities of untargeted metabolites in IPTH-induced *B. subtilis* populations. BsMDH/inactBsMDH, active/inactive *B. subtilis* MDH; StMDH/inactStMDH, active/inactive *S. sciuri* MDH*;* OceMDH/inactOceMDH, active/inactive *O. iheyensis* MDH; control, *B. subtilis* carrying empty pHT254 vector. Source data to generate this figure are provided as a Supplementary Data file 4.

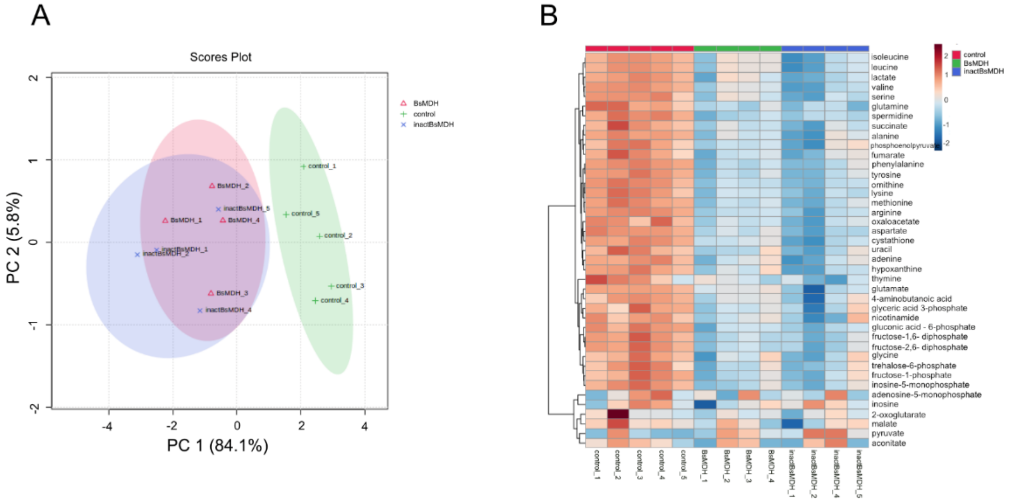

Supplementary Figure 9**. (A)** PCA of targeted metabolite intensities identified in four *B. subtilis* replicate populations expressing either active or inactive endogenous and orthologous MDH proteins and control populations from IPTG-induced pHT254 vector. Note the separation on the PC1 axis between *B. subtilis* replicate populations expressing active/inactive endogenous MDH proteins and the rest of the populations. **(B)** Heat map of -$\log_2$ of relative intensities of targeted metabolites in IPTH-induced *B. subtilis* populations. BsMDH/inactBsMDH, active/inactive *B. subtilis* MDH; control, *B. subtilis* carrying empty pHT254 vector. Source data to generate this figure are provided as a Supplementary Data file 3.

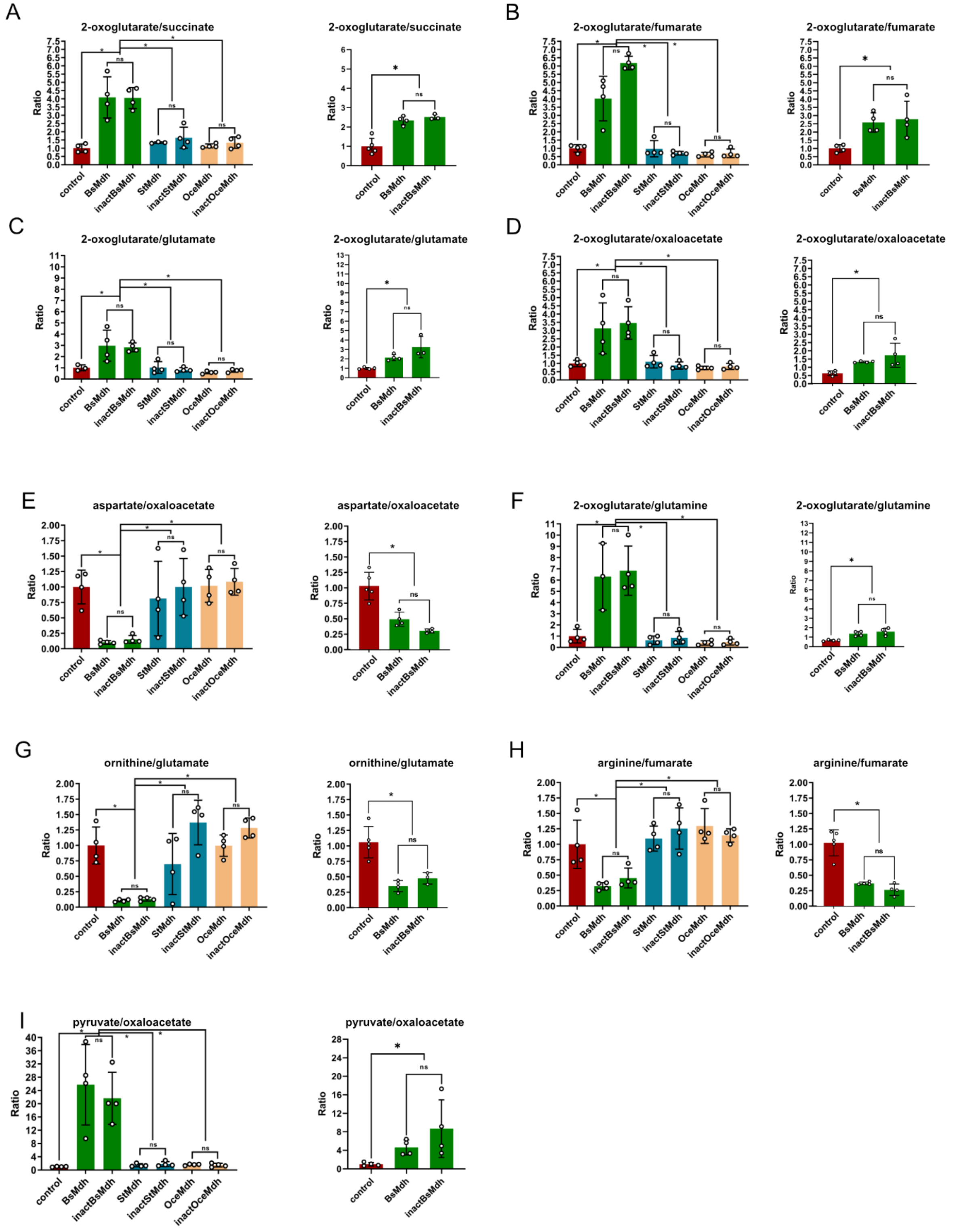

Supplementary Figure 10**. Comparison of changes in metabolite ratios between two replicate experiments. (A-I)** *Left panel*. Data are related to the experimental dataset presented in Fig. 3E (main text).

Right panel. Data are related to the experimental dataset presented in Supplementary Fig. 9. Histograms represent mean values ± SD from four independent biological replicates (*n* = 4). Individual values are shown as empty circles. Note the similarity in the ratio patterns between the two experiments. Asterisks indicate significant differences (p = 0.029, two-tailed Mann Whitney test). ns, non-significant. BsMDH/inactBsMDH, active/inactive *B. subtilis* MDH; StMDH/inactStMDH, active/inactive *S. sciuri* MDH*;* OceMDH/inactOceMDH, active/inactive *O. iheyensis* MDH; control, *B. subtilis* carrying empty pHT254 vector. Source data to generate this figure are provided as a Source Data file.

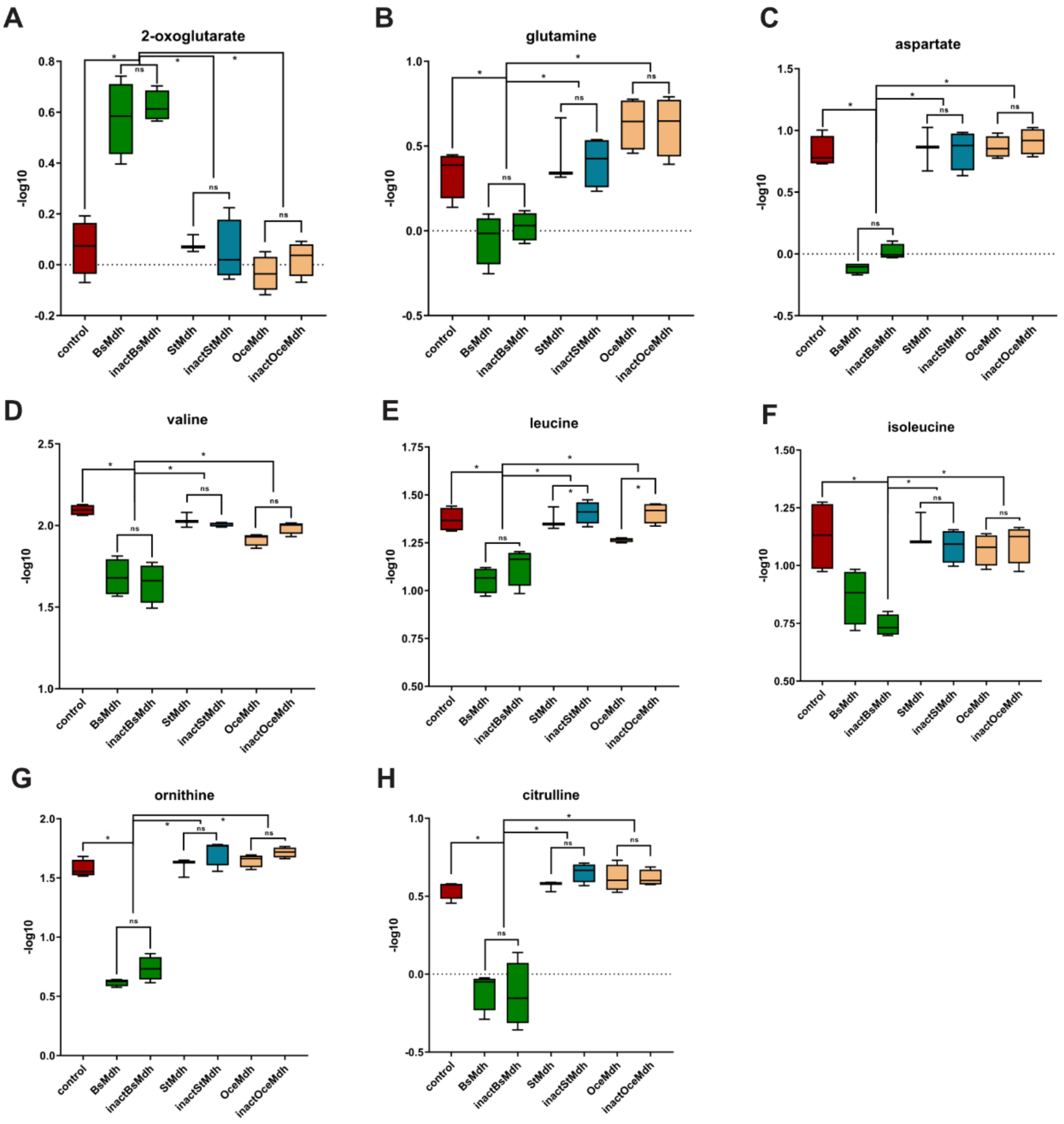

Supplementary Figure 11**. Relative intensities of the identified metabolites upon MDH overexpression. (A-H)** -log10 of intensities of individual metabolites. Boxes represent tenth to ninetieth percentile. Horizontal bars represent mean values from three independent measurements (*n* = 3). Whiskers represent the spread of individual values from minimum to maximum. Asterisks indicate significant differences (p = 0.029, two-tailed Mann Whitney test). ns, non-significant. BsMDH/inactBsMDH, active/inactive *B. subtilis* MDH; StMDH/inactStMDH, active/inactive *S. sciuri* MDH*;* OceMDH/inactOceMDH, active/inactive *O. iheyensis* MDH; control, *B. subtilis* carrying empty pHT254 vector. Source data to generate this figure are provided as a Source Data file.

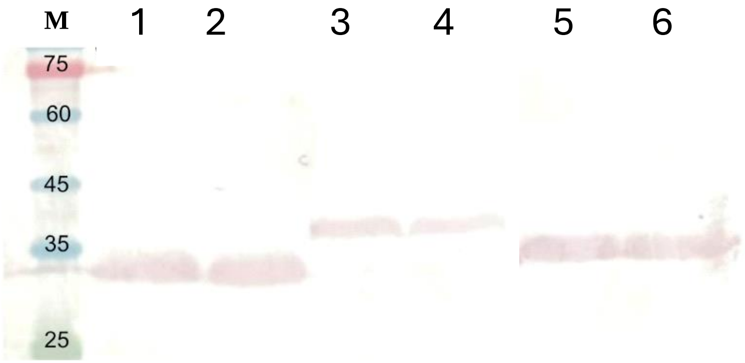

Supplementary Figure 12**. Western blot analysis of MDH expression levels in lysates of cultures propagated on glutamine as the main carbon source.** 1,2 **-** active, inactive *S. sciuri* MDH*;* 2,3 – active, inactive *O. iheyensis* MDH; 3,4 – active, inactive *B. subtilis* MDH. The detection was done with anti-His antibodies. M, protein marker, kDa. Source data to generate this figure are provided as a Source Data file.

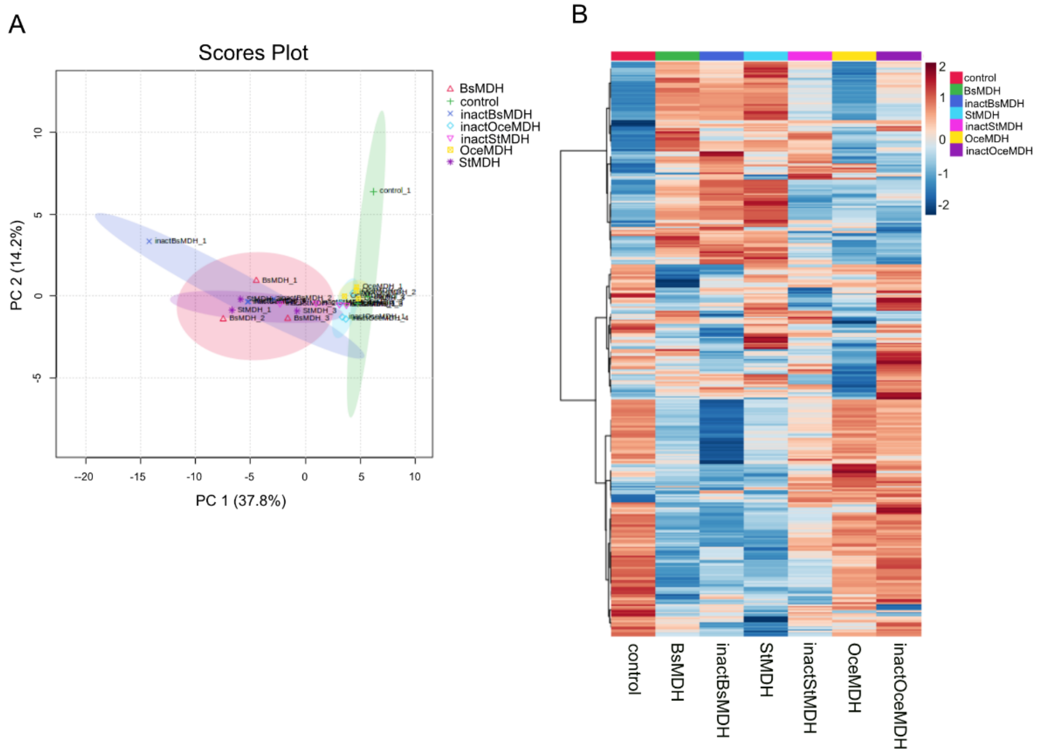

Supplementary Figure 13**. (A)** PCA of untargeted metabolite intensities identified in four *B. subtilis* replicate populations expressing either active or inactive endogenous and orthologous MDH proteins and control populations carrying empty pHT254 vector. The cultures were propagated on glutamine as the main carbon source and induced with IPTG. **(B)** Heat map of -$\log_2$ of relative intensities of untargeted metabolites. BsMDH/inactBsMDH, active/inactive *B. subtilis* MDH; StMDH/inactStMDH, active/inactive *S. sciuri* MDH*;* OceMDH/inactOceMDH, active/inactive *O. iheyensis* MDH; control, *B. subtilis* carrying empty pHT254 vector. Source data to generate this figure are provided as a Supplementary Data file 6.

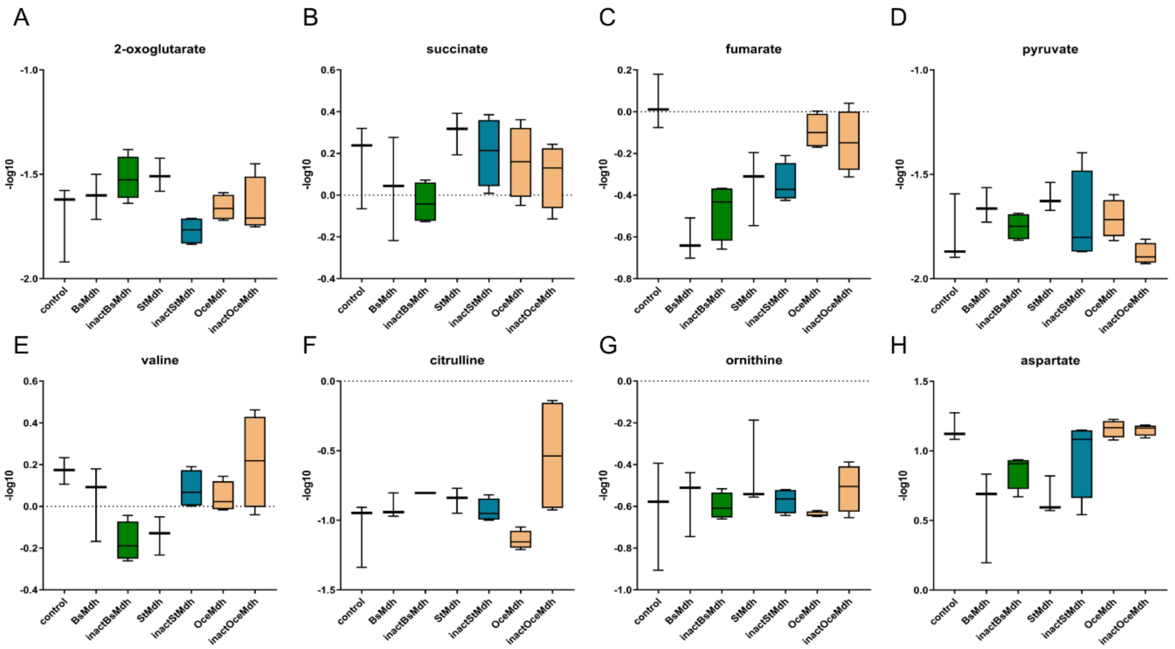

Supplementary Figure 14**. Relative intensities of the identified metabolites upon MDH overexpression with glutamine as the main carbon source. (A-H)** -log10 of intensities of individual metabolites. Boxes represent tenth to ninetieth percentile. Horizontal bars represent mean values from three independent measurements (*n* = 3). Whiskers represent the spread of individual values from min to max. No significant changes between strains can be found (p = 0.9, two-tailed Mann Whitney test). BsMDH/inactBsMDH, active/inactive *B. subtilis* MDH; StMDH/inactStMDH, active/inactive *S. sciuri* MDH*;* OceMDH/inactOceMDH, active/inactive *O. iheyensis* MDH; control, *B. subtilis* carrying empty pHT254 vector. BsMDH/inactBsMDH, active/inactive *B. subtilis* MDH; control, *B. subtilis* carrying empty pHT254 vector. Source data to generate this figure are provided as a Source Data file.

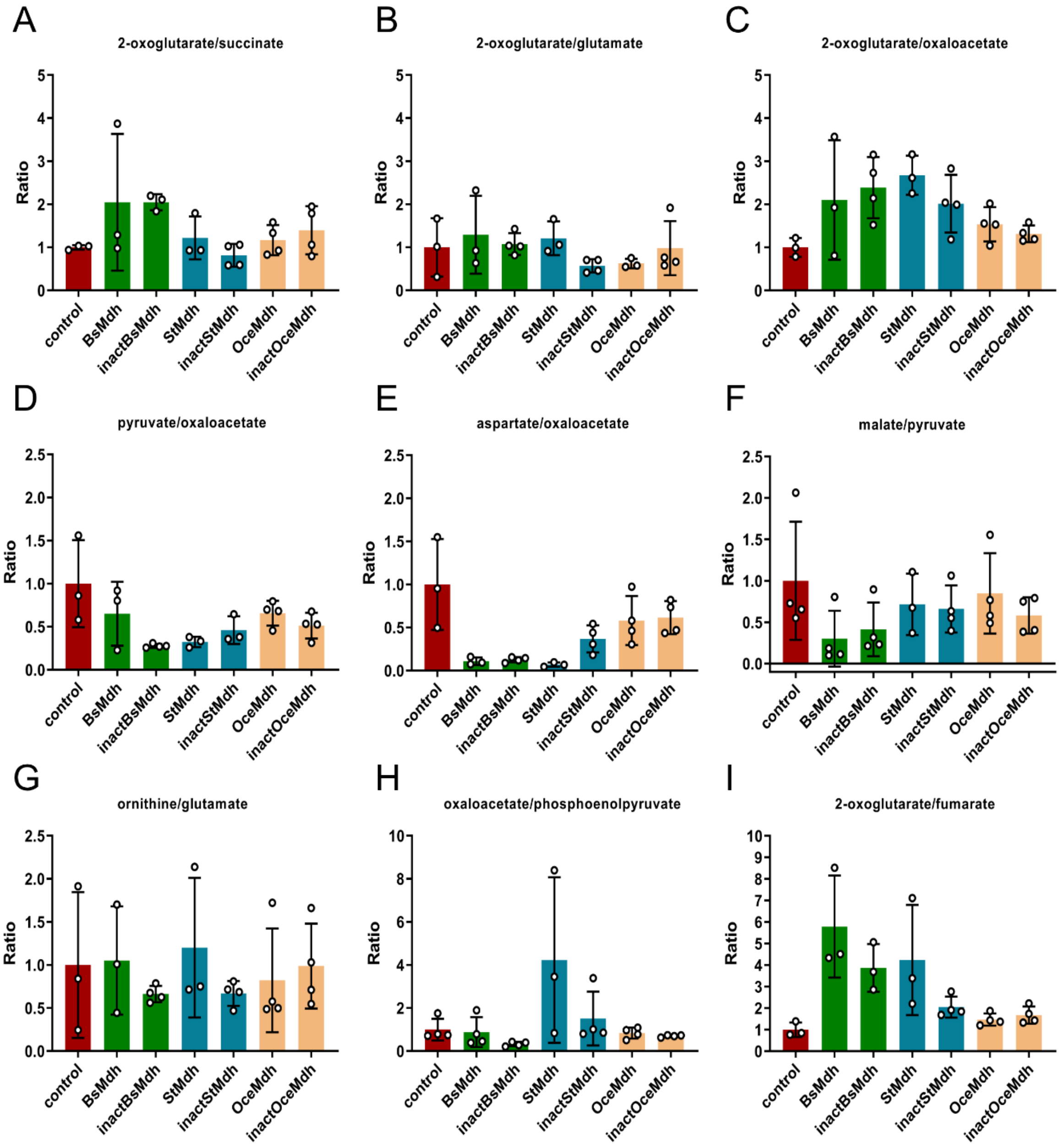

Supplementary Figure 15**. (A-I) Ratios of relative intensities of targeted metabolites in propagated on glutamine as the main carbon source.** Histograms represent mean values ± SD from four independent biological replicates (*n* = 4). Individual values are shown as empty circles. BsMDH/inactBsMDH, active/inactive *B. subtilis* MDH; StMDH/inactStMDH, active/inactive *S. sciuri* MDH*;* OceMDH/inactOceMDH, active/inactive *O. iheyensis* MDH; control, *B. subtilis* carrying empty pHT254 vector. No significant differences in metabolite ratios between strains can be detected (p = 0.9, two-tailed Mann Whitney test). Source data to generate this figure are provided as a Source Data file.

A B

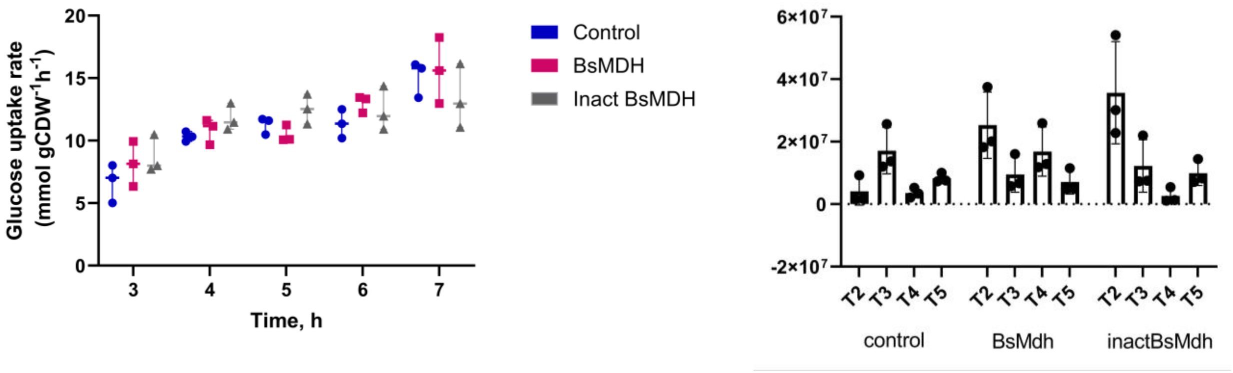

Supplementary Figure 16**. Determination of metabolic steady state in ^{13}C-tracer experiments**. **(A)** Glucose consumption rate measured at 3 (T1), 4 (T2), 5 (T3), 6 (T4), 7 (T5) hours of growth. The steady levels of glucose consumption are clearly apparent at T2-T4. Horizontal bars represent mean values of three independent experiments (*n* = 3), whiskers indicate the spread of individual values from min to max. Individual values are shown as circles (control), squares (BsMDH), and triangles (Inact BsMDH). **(B)** Relative intensities of sum of all targeted metabolites per sample normalized by cell dry mass are shown at time points as in (A). Histograms represent mean values ± SD from 3 independent biological replicates (*n* = 3). Individual values are shown as circles. No significant difference between normalized metabolite levels can be seen between individual time points, thus indicating metabolic steady state (p = 0.9, two-tailed Mann Whitney test). BsMDH/inactBsMDH, active/inactive *B. subtilis* MDH; control, *B. subtilis* carrying empty pHT254 vector. Source data to generate this figure are provided as a Source Data file.

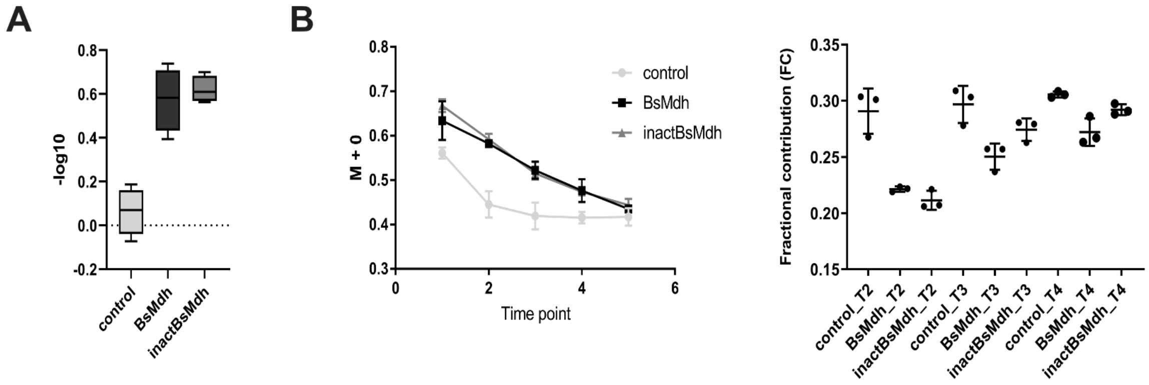

Supplementary Figure 17**. ^{13}C tracer analysis of 2-oxoglutarate metabolic flux. (A)** -log10 of the relative intensities of 2-oxoglutarate in *B. subtilis* strain expressing active and inactive endogenous MDH (BsMDH and inactBsMDH, respectively) from IPTG-induced pHT254 vector or carrying an empty vector (control). Boxes represent tenth to ninetieth percentile. Horizontal bars represent mean values from three independent measurements (n = 3). Whiskers represent the spread of individual values from minimum to maximum. **(B)** *Left panel*. Drop in fractional abundance of unlabeled isotopologues (M+0) due to labeling with heavy isotope of glucose (^{13}C). The drop might be a result of an increase in 2-oxoglutarate pool and not a reduction in metabolic flux. Time points 2-4 correspond to metabolic steady state. Data are represented as mean values ± SD from three independent experiments (n = 3). *Right panel*. Fractional contribution of the fully ^{13}C-labeled metabolite. Time points T2-T4 correspond to metabolic steady state. Horizontal bars represent mean values for three independent experiments (n = 3). Vertical bars indicate ± SD. Individual values are shown as dots. Source data to generate this figure are provided as a Source Data file.

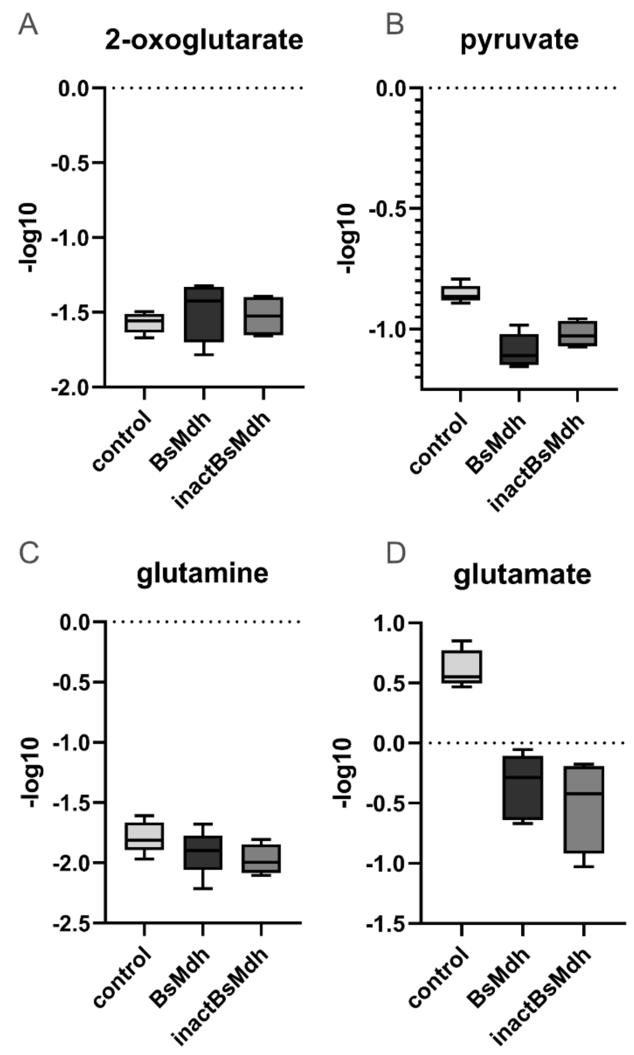

Supplementary Figure 18**. Relative intensities of the identified metabolites upon MDH overexpression in the presence of dimethyl-2-oxoglutarate. (A-D)** -log10 of intensities of individual metabolites in *B. subtilis* strain expressing active and inactive endogenous MDH (BsMDH and inactBsMDH, respectively) from IPTG-induced pHT254 vector or carrying an empty vector (control). Horizontal bars represent mean values from three independent measurements (n = 3). Whiskers represent the spread of individual values from minimum to maximum. Source data to generate this figure are provided as a Source Data file.

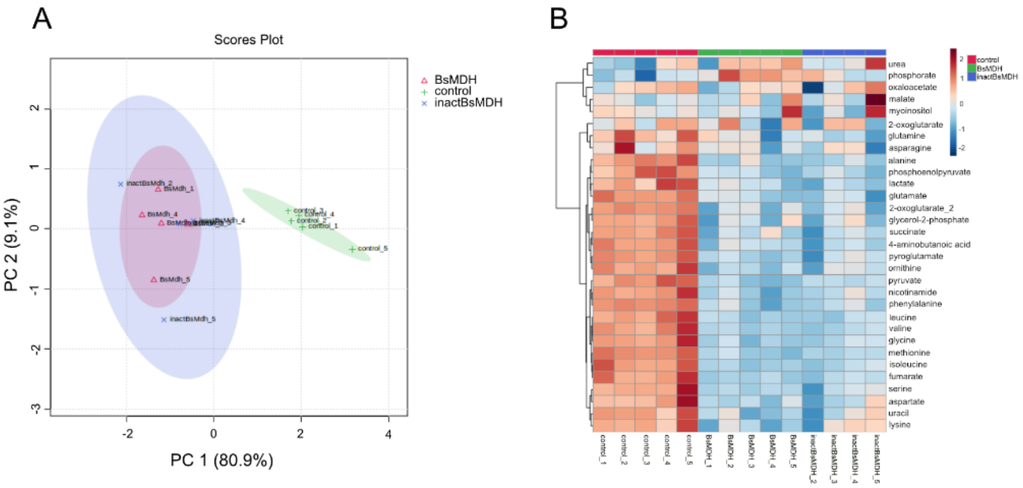

Supplementary Figure 19**. (A)** PCA of targeted metabolite intensities identified in four *B. subtilis* replicate populations expressing either active or inactive endogenous and orthologous MDH proteins and control populations from IPTG-induced pHT254 vector in the presence of dimethyl-2-oxoglutarate. Note the separation on the PC1 axis between *B. subtilis* replicate populations expressing active/inactive endogenous MDH proteins and the rest of the populations. **(B)** Heat map of -$\log_2$ of relative intensities of targeted metabolites in IPTH-induced *B. subtilis* populations. BsMDH/inactBsMDH, active/inactive *B. subtilis* MDH; control, *B. subtilis* carrying empty pHT254 vector. Source data to generate this figure are provided as a Supplementary Data file 7.

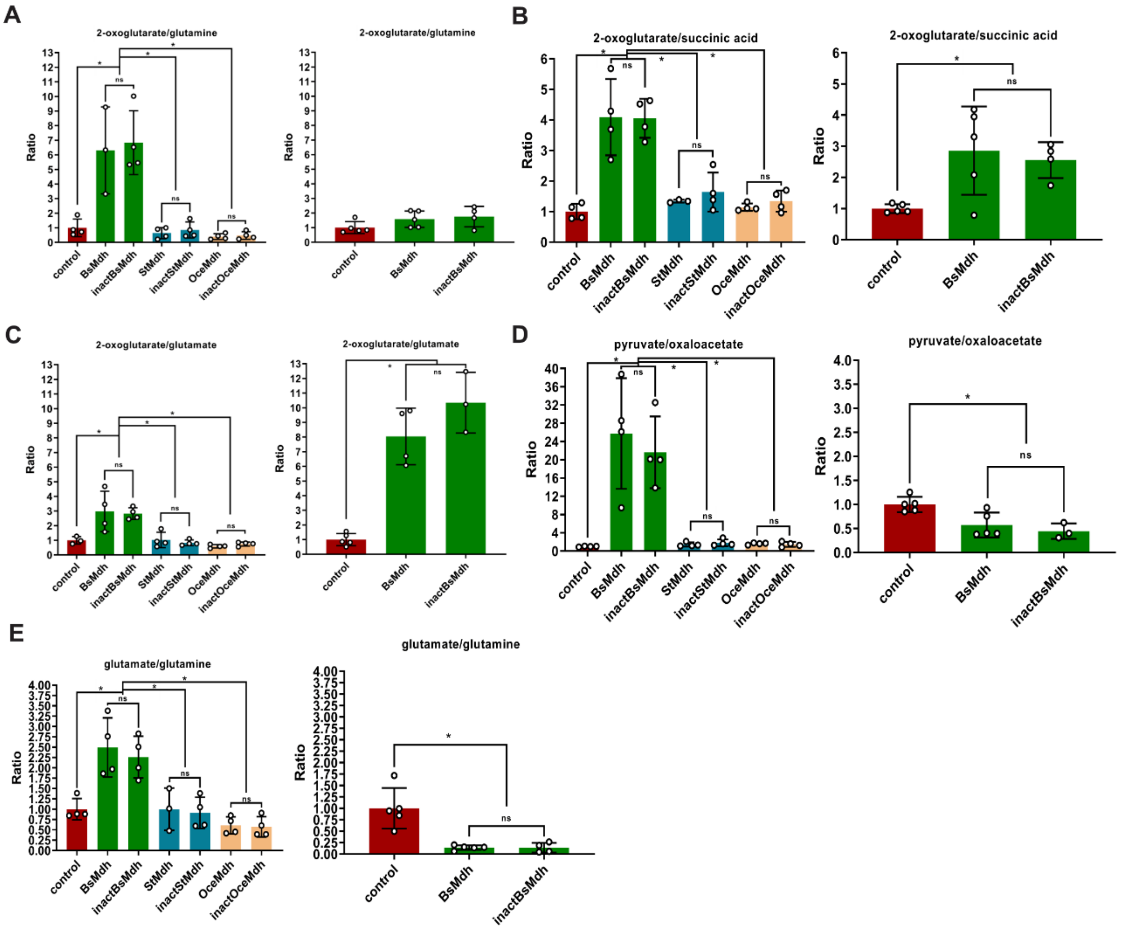

Supplementary Figure 20**. (A-E) The effect of MDH overexpression on metabolic ratios in the presence of 2-methyl-oxoglutarate.** *Left panels*. Changes in metabolic ratios of individual metabolites of B. subtilis strains overexpressing MDH and grown in the presence of glucose and ammonium *Right panels*. Same as left panels, but with addition of dimethyl-oxoglutarate to the growth medium. Histograms represent mean values ± SD from four independent biological replicates (n = 4). Individual values are shown as empty circles. Asterisks indicate significant differences (p = 0.029, two-tailed Mann Whitney test). ns, non-significant. BsMDH/inactBsMDH, active/inactive *B. subtilis* MDH; StMDH/inactStMDH, active/inactive *S. sciuri* MDH*;* OceMDH/inactOceMDH, active/inactive *O. iheyensis* MDH; control, *B. subtilis* carrying empty pHT254 vector. Source data to generate this figure are provided as a Source Data file.

Supplementary Table 1. % Amino acid sequence identity of orthologous MDH proteins relative to *B. subtilis's* MDH for a representative set of bacterial MDH proteins depicted in Supplementary Fig. 2.

	% identity	Phylum	Class	Order
Geobacillus kaustophilus	86	Firmicutes	Bacilli	Bacillales
Oceanobacillus iheynesis	82	Firmicutes	Bacilli	Bacillales
Halobacillus halophilus	81	Firmicutes	Bacilli	Bacillales
Brevibacillus brevis	78	Firmicutes	Bacilli	Bacillales
Staphylococcus sciuri	76	Firmicutes	Bacilli	Bacillales
Exiguobacterium antarticum	75	Firmicute	Bacilli	Bacillales
Paenibacillus polymyxa	72	Firmicutes	Bacilli	Bacillales
Brucella suis	53	Proteobacteria	Alpha	Rhizobiales
Geobacter metallireducens	52	Proteobacteria	Delta	Desulfromonadales
Aquifex aeolicus	50	Aquificae	Aquificae	Aquificales
Francisella tularensis	49	Proteobacteria	Gamma	Thiotricales
Wolinella succinogenes	42	Proteobacteria	Epsilon	Campylobacterales
Megamonas funiformis	41	Firmicutes	Negativicutes	Selenomonadales
Listeria monocytogenes	37	Firmicutes	Bacilli	Bacillales
Thermotoga maritima	36	Thermotogae	Thermotogales	Thermotogaceae
Pasteurella multocida	31	Proteobacteria	Gamma	Pasteuellales
Aeromonas hydrophila	31	Proteobacteria	Gamma	Aeromonadales
Escherichia coli	31	Proteobacteria	Gamma	Enterobacteriaea
Vibrio cholera	30	Proteobacteria	Gamma	Vibrionales
Clostridium botulinum	26	Firmicutes	Clostridia	Clostridiales

Supplementary Table 2. Kinetic parameters of orthologous MDH proteins

MDH ortholog	aa seq identity,%	k_{cat}, sec^{-1}	K_M (oxaloacetate), μM	k_{cat}/K_M, $sec^{-1}μM^{-1}$
Bacillus subtilis	100	120	12	10
Geobacillus kaustophilus	86	59	54	1.1
Oceanobacillus iheynesis	82	249	23	10.8
Staphylococcus sciuri	76	188	22	8.5
Aquifex aeolicus	50	25	21	1.2
Escherichia coli	31	102	39	2.6

Supplementary Table 3. Parameters used in cluster-mediated substrate channeling model.

**Parameters**	**Comments**
**Radius length, R (μm)**	
R_1 = 5 (cluster within droplet); R_1 = 0.1(cluster within droplets)	Adjustable parameter
R_2 = 10 (droplet); R_2= 2 (bacterial cell)	Experimentally determined. See (Fig. S4 and http://book.bionumbers.org)
Basin R_0 = 18 (LLPS); R_0 = 3.65 (bacterial cell)	See **Supplementary Appendix** for detailed calculations
**Diffusion rate, *D* (μm²/sec)**	
$D = 15$	Estimated from http://book.bionumbers.org
**Product degradation rate, β (sec^{-1})**	
$\beta = 0.075$	Adjustable parameter. The ratio of β and c^* determines the order-of-magnitude correspondence between theory and experiment for the units of the product and input (substrate) metabolites (See **Fig. 2**)
**Rate of relaxation of input metabolite to homeostatic concentration level, α_0 (sec^{-1})**	
$\alpha_0 = 1$	Taken from Castellana et al. Nat Biotech 2014.
**Substrate concentration at homeostasis, c_0^* (mM)**	
$c_0^* = 0.0055$	See note on β
**Enzyme concentration (mM)**	
N_{total} = 0.00012 (ICD concentration within droplets)	Experimentally determined (see **Materials and Methods**)
**Activity, k_{cat}/K_M (mM^{-1}sec^{-1})**	
$k = k_{cat}/K_M$ = 10 000	https://www.brenda-enzymes.org/enzyme.php?ecno=1.1.1.42#ORGANISM

Supplementary Notes

Detailed theory

Part 1: Solution to the reaction-diffusion equation

Assuming steady state, that is, no time-dependence for the concentration of the substrate $c_0(\vec{\mathbf{r}})$ and the product $c_1(\vec{\mathbf{r}})$, the Equations 4 and 5 (main text) are reduced to:

$$0 = D\nabla^2 c_0(\vec{\mathbf{r}}, t) \; - k\, n(\vec{\mathbf{r}})\, c_0(\vec{\mathbf{r}}, t) - \alpha_0(c_0(\vec{\mathbf{r}}, t) - c_0^*) \qquad (1)$$

$$0 = D\nabla^2 c_1(\vec{\mathbf{r}}, t) \; + k\, n(\vec{\mathbf{r}})\, c_0(\vec{\mathbf{r}}, t) - \beta c_1(\vec{\mathbf{r}}, t) \qquad (2)$$

Furthermore, without loss of generality and due to the spherical symmetry of the model in **Fig. 2** (main text), Equations 1 and 2, can be recast only due to their radial dependence:

$$0 = D\frac{\partial^2(r\, c_0(r))}{\partial r^2} - k\, n(r)\, c_0(r) - \alpha_0(c_0(r) - c_0^*) \qquad (3)$$

$$0 = D\frac{\partial^2(r\, c_1(r))}{\partial r^2} + k\, n(r)\, c_0(r) - \beta c_1(r) \qquad (4)$$

For mathematical convenience, we perform a change of variables, $\phi_i(r) \equiv r c_i(r)$, where $i = 0{,}1$ subscripts correspond to the substrate and the product.

$$0 = D\frac{\partial^2(\phi_0(r))}{\partial r^2} - k\, n(r)\phi_0(r) - \alpha_0(\phi_0(r) - r\, c_0^*) \qquad (5)$$

$$0 = D\frac{\partial^2(\phi_1(r))}{\partial r^2} + k\, n(r)\, \phi_0(r) - \beta\phi_1(r) \qquad (6)$$

These differential equations can be written more explicitly for each corresponding volume in Fig. 2, where the enzyme concentrations are pre-defined.

Inside the cluster, $r \leq R_1$: Volume v_1, with the effective enzyme activity $a_{cluster}$ (Equation 5, main text):

$$0 = D\frac{\partial^2(\phi_{0,cluster}(r))}{\partial r^2} - a_{cluster}\phi_{0,cluster}(r) - \alpha_0\left(\phi_{0,cluster}(r) - r\, c_0^*\right) \qquad (7)$$

$$0 = D\frac{\partial^2(\phi_{1,cluster}(r))}{\partial r^2} + a_{cluster}\, \phi_{0,cluster}(r) - \beta\phi_{1,cluster}(r) \qquad (8)$$

Inside the droplet but outside the cluster, $R_1 \leq r \leq R_2$: Volume v_2, with the effective enzyme activity $a_{cluster}$ (Equation 4, main text):

$$0 = D\frac{\partial^2(\phi_{0,drop}(r))}{\partial r^2} - a_{drop}\phi_{0,drop}(r) - \alpha_0\left(\phi_{0,drop}(r) - r\, c_0^*\right) \qquad (9)$$

$$0 = D\frac{\partial^2(\phi_{1,drop}(r))}{\partial r^2} + a_{drop}\,\phi_{0,drop}(r) - \beta\phi_{1,drop}(r) \tag{10}$$

Bulk (outside the droplet), $R_2 \leq r \leq R_0$: Volume v_0, with enzyme concentration zero

$$0 = D\frac{\partial^2(\phi_{1,bulk}(r))}{\partial r^2} - \beta\phi_{1,bulk}(r) \tag{11}$$

The constants of integration arising from Equations 1-11 can be determined by imposing boundary conditions. Specifically, for the substrate, we impose its continuity and that of its derivative at the cluster-drop interface:

$$\phi_{0,cluster}(r)|_{r=R_1} = \phi_{0,drop}(r)|_{r=R_1} \tag{12}$$

$$\frac{\partial}{\partial r}\phi_{0,cluster}(r)|_{r=R_1} = \frac{\partial}{\partial r}\phi_{0,drop}(r)|_{r=R_1}$$

as well as the drop-bulk interface

$$\phi_{0,drop}(r)|_{r=R_2} = \phi_{0,bulk}(r)|_{r=R_2} \tag{13}$$

$$\frac{\partial}{\partial r}\phi_{0,drop}(r)|_{r=R_2} = \frac{\partial}{\partial r}\phi_{0,bulk}(r)|_{r=R_2}$$

For the product, we impose similar boundary conditions:

$$\phi_{1,cluster}(r)|_{r=R_1} = \phi_{1,drop}(r)|_{r=R_1} \tag{14}$$

$$\frac{\partial}{\partial r}\phi_{1,cluster}(r)|_{r=R_1} = \frac{\partial}{\partial r}\phi_{1,drop}(r)|_{r=R_1}$$

as well as the drop-bulk interface

$$\phi_{1,drop}(r)|_{r=R_2} = \phi_{1,bulk}(r)|_{r=R_2} \tag{15}$$

$$\frac{\partial}{\partial r}\phi_{1,drop}(r)|_{r=R_2} = \frac{\partial}{\partial r}\phi_{1,bulk}(r)|_{r=R_2}$$

Since the concentration of the product at the interface of all the basins should be constant,

$$\frac{\partial}{\partial r}\phi_{1,drop}(r)|_{r=R_0} = 0 \tag{16}$$

Part 2: Calculation of effective basin radius, R_0

The mean size of the droplets for *in vitro* LLPS experiments was measured to be around 10 mm (**Supplementary Fig. 4, Supplementary Table 3**). The fraction of volume ρ occupied by the droplet phase (all the droplets in the system) versus the supernatant (i.e., continuous phase) is

$v_{supernatant} = v_{basin} - v_{drop}$.

Thus,

$$\rho = \frac{v_{drop}}{v_{supernatant}} = \frac{v_{drop}}{v_{basin} - v_{drop}} = \frac{\frac{4}{3}\pi R_2^3}{\frac{4}{3}\pi R_0^3 - \frac{4}{3}\pi R_2^3} \qquad (17)$$

or

$$R_0 = R_2 \sqrt[3]{\frac{1}{\rho} + 1}$$

For the droplet size of 10 mm and a volume fraction of 20% for the droplets ($\rho = 0.2$), the radius of the basin is ~18 mm.
